# Minimally Invasive and Emerging Diagnostic Approaches in Endometrial Cancer: Epigenetic Insights and the Promise of DNA Methylation

**DOI:** 10.3390/diagnostics14222575

**Published:** 2024-11-15

**Authors:** Floriana Porcaro, Antonella Paolucci, Piercarmine Porcaro, Gaetano Cardinale, Antonia Romitelli, Domenico Cozzolino, Serena Voccola

**Affiliations:** 1 Centro Medico Delta S.r.l., 82030 Apollosa, Italy; floriana.porcaro@gmail.com (F.P.); piercarmine.porcaro1@gmail.com (P.P.); 2 Centro Prevenzione Donna, 05100 Terni, Italy; antonellapao@icloud.com; 3 Tecno Bios S.r.l., 82030 Apollosa, Italy; gaetano.cardinale@tecnobios.com (G.C.); domenico.cozzolino@tecnobios.com (D.C.); 4 Delta Biotech S.r.l., 82030 Apollosa, Italy; antonia.romitelli@deltabiotech.it; 5 Consorzio Sannio Tech, 82030 Apollosa, Italy

**Keywords:** endometrial cancer, DNA methylation, epigenetic biomarkers, non-invasive diagnostics, obesity-related cancer risk, early cancer detection

## Abstract

Endometrial cancer (EC) is the most common gynecological malignancy, with rising incidence and mortality rates. Key risk factors, including obesity, prolonged estrogen exposure, and metabolic disorders, underscore the urgent need for non-invasive, early diagnostic tools. This review focuses on the role of DNA methylation as a potential biomarker for early EC detection. Aberrant DNA methylation in the promoter regions of tumor suppressor genes can lead to gene silencing and cancer progression. We examine recent studies utilizing minimally invasive samples, such as urine, cervicovaginal, and cervical scrapes, to detect early-stage EC through DNA methylation patterns. Markers such as RASSF1A, HIST1H4F, GHSR, SST, and ZIC1 have demonstrated high diagnostic accuracy, with AUC values up to 0.95, effectively distinguishing EC from non-cancerous conditions. This review highlights the potential of DNA methylation-based testing as a non-invasive alternative to traditional diagnostic methods, offering earlier detection, better risk stratification, and more personalized treatment plans. These innovations hold the promise of transforming clinical practice by enabling more timely and effective management of endometrial cancer.

## 1. Introduction

### Epidemiology and Risk Factors of Endometrial Carcinoma

Endometrial cancer (EC) is a malignancy of the inner epithelial lining of the uterus. It is the most prevalent gynecological malignancy, with global incidence and mortality rates on the rise [1]. Most women are between 65 and 75 years old when they are diagnosed with endometrial cancer [2]. The increasing incidence of endometrial cancer represents a significant concern for public health, especially in developed countries. Recent studies have projected a considerable rise in the incidence of this cancer in the coming decades. Specifically, the best-fitting model projected an increase to 42.13 endometrial cancer cases per 100,000 by the year 2030, a 55% increase over 2010 rates [3]. These rising projections underscore the urgent need for more efficient and less invasive diagnostic tools, which could facilitate early detection and improved patient outcomes.

The concerning statistics on the incidence and mortality of endometrial cancer, along with unsettling future projections, highlight the urgent need to develop innovative diagnostic methods and biomarkers that can expedite the diagnosis and prognosis of patients [4].

Endometrial cancer primarily manifests through symptoms such as abnormal vaginal bleeding, especially in postmenopausal women, a common and early signal [2,5]. Given that early detection is closely linked to more favorable prognosis, there is a growing emphasis on developing biomarkers and diagnostic methods that are both accurate and accessible. In premenopausal women, irregular or heavy menstruation may indicate the disease, while other symptoms include pelvic pain, dyspareunia, and abnormal vaginal discharge. The increase in the incidence of endometrial cancer is largely due to a rise in various risk factors within the general population. The rise in EC incidence is largely attributed to an increase in various risk factors, with obesity being one of the most significant independent risk factors, as it increases EC risk more than other cancers [5,6,7]. Other risk factors include early menarche, late menopause, hyperinsulinemia, insulin resistance, and hypertension. Prolonged estrogen exposure, whether through tamoxifen use or unopposed estrogen therapy, as well as nulliparity and polycystic ovary syndrome (PCOS), also contribute to elevated risk. Metabolic conditions such as diabetes and genetic predispositions, including Lynch syndrome, further emphasize the interplay between environmental and genetic influences in EC etiology [3]. These genetic and environmental factors also influence epigenetic changes, such as DNA methylation patterns, which can further exacerbate cancer risk.

The prognosis for endometrial cancer is favorable in the early stages when the tumor is confined to the endometrium. Patients with stage I (localized) endometrial cancer have a 5-year survival rate of 80–90%. However, for those diagnosed at stage III, the 5-year survival rate decreases to 50–65%, and for stage IV, it further drops to 15–17% [8].

Early diagnosis is, therefore, crucial for improving outcomes. Identifying EC in its early stages can reduce the need for invasive surgeries or additional treatments, resulting in lower healthcare costs, reduced complications, and improved survival rates [9]. In light of these benefits, the current review explores recent advancements in minimally invasive biomarkers for EC, particularly DNA methylation markers that offer promise for early detection. Recent advances in cancer biology have revealed that epigenetic modifications, particularly DNA methylation, play a pivotal role in the initiation and progression of EC. These heritable changes in gene expression do not alter the underlying DNA sequence but are influenced by both genetic and environmental factors, such as obesity, diabetes, and prolonged estrogen exposure [10]. One of the most significant epigenetic changes in EC is the aberrant methylation of DNA in the promoter regions of tumor suppressor genes, which can lead to gene silencing and unchecked cell proliferation [11]. Factors such as insulin resistance and hormonal imbalances further modulate these epigenetic changes, exacerbating cancer risk. These DNA methylation changes, occurring early in carcinogenesis, present a unique opportunity for developing non-invasive diagnostic tests capable of identifying cancer before symptoms manifest. DNA methylation patterns have emerged as promising biomarkers for the early detection of EC. Since aberrant methylation occurs early in carcinogenesis, it offers a unique opportunity to develop non-invasive diagnostic tests capable of detecting cancer at its earliest stages, even before symptoms manifest. This connection between established risk factors and epigenetic changes presents novel diagnostic approaches, potentially enabling earlier detection, better risk stratification, and more personalized treatment plans, ultimately improving patient outcomes [12]. Recent advancements in cancer biology have highlighted the importance of epigenetic modifications, particularly DNA methylation, in the pathogenesis of endometrial cancer. While previous works have provided valuable insights into biopsy-based diagnostic approaches and specific panels of epigenetic markers, this review emphasizes the latest advancements in minimally invasive methods and self-sampling techniques for endometrial cancer detection. Covering literature from the past decade (2013–2023), this work focuses on DNA methylation biomarkers that can be detected in cervicovaginal fluid and urine, aiming to propose these as non-invasive alternatives to traditional biopsies. By synthesizing recent literature, this review aims to provide a comprehensive overview of the advancements in non-invasive epigenetic biomarkers, underscoring their potential for clinical applications in early EC detection.

## 2. Histological and Molecular Classification of Endometrial Cancer

EC is a heterogeneous disease [13]. Historically, it was classified according to Bokhman’s classification [14], which divides EC into two types: Type I (endometrioid carcinoma), typically associated with obesity and prolonged estrogen exposure; and Type II, which includes several histological subtypes such as clear cell carcinoma, serous carcinoma, and less common variants [3]. A 2013 study suggests that both Type I and Type II endometrial cancers share several etiological factors, indicating that Type II tumors may not be entirely independent of estrogen, contrary to previous assumptions [15].

Type II endometrial tumors tend to have worse prognoses and are typically less differentiated. Although they account for only 10–20% of all endometrial cancer cases, Type II tumors are disproportionately lethal, contributing to approximately 40% of endometrial cancer-related deaths [16].

While histological classification has long been central to diagnosing EC, the introduction of molecular classification through The Cancer Genome Atlas (TCGA) project has revolutionized the understanding of tumor biology. Molecular classification provides deeper insights into tumor behavior, enhancing prognostic stratification and enabling more personalized therapeutic approaches [17]. From a molecular standpoint, EC is now classified into four distinct subtypes, as defined by the TCGA project:

- POLE-ultramutated: Characterized by mutations in the POLE gene, which encodes DNA polymerase epsilon, a critical enzyme in DNA replication and repair. These tumors exhibit an exceptionally high mutational burden and are associated with a favorable prognosis.
- Microsatellite Instability-High (MSI-H): Tumors in this group show defects in the DNA mismatch repair (MMR) pathway, leading to microsatellite instability. In many cases, this instability is caused by the methylation of the MLH1 promoter, which silences this critical repair gene. These tumors tend to have an intermediate prognosis and may respond well to immunotherapy, particularly immune checkpoint inhibitors.
- Copy-Number Low (CNL): Also referred to as TP53 wild-type or “no specific molecular profile” (NSMP), these tumors exhibit low levels of genomic alterations and typically show endometrioid histology. Their prognosis is intermediate. Emerging studies suggest that specific DNA methylation profiles within this group could provide additional prognostic and therapeutic insights.
- Copy-Number High (CNH): This subtype is characterized by widespread genomic instability, including TP53 mutations and multiple copy-number alterations. It is often associated with serous histology and has a poorer prognosis due to its aggressive behavior. In this group, DNA methylation analysis may reveal additional epigenetic biomarkers relevant to tumor behavior.

## 3. Diagnostic Approaches for Endometrial Carcinoma

Early diagnosis is essential for improving the prognosis of endometrial carcinoma (EC), making the selection and sequence of diagnostic tests crucial. Unlike colorectal cancer, where screening tools like the SOF or FIT are available, EC lacks a standardized screening test. As a result, patients typically undergo second-level tests only after presenting symptoms such as abnormal uterine bleeding or persistent menstrual irregularities. Current screening techniques include transvaginal ultrasound (TVU), hysteroscopy, and endometrial biopsy, along with advanced imaging modalities like computed tomography (CT) and magnetic resonance imaging (MRI). TVU is a well-tolerated, non-invasive method that can identify abnormalities such as endometrial thickening, cysts, fluid in the uterine cavity, or polyps, all of which are associated with an increased risk of EC [18]. However, like all ultrasound-based techniques, the effectiveness of TVU depends on the operator’s skill [19], and the presence of adipose tissue can degrade image quality [18], making it less reliable in women with high BMI [20]

In postmenopausal women, an endometrial thickness threshold of 5 mm detected through transvaginal ultrasound offers a sensitivity of 96.2% for diagnosing endometrial cancer and a high negative predictive value of 99.3%, according to a large systematic review of 44 studies involving 1341 cases and 15,998 controls [21]. However, the specificity of this criterion is limited, around 51.5%, meaning that a significant number of women require further diagnostic investigations to definitively rule out significant endometrial pathology. In premenopausal women, TVU’s specificity is further reduced due to cyclical fluctuations in endometrial thickness during the menstrual cycle, often necessitating additional tests to reach an accurate diagnosis [22].

While TVUS is effective in detecting structural abnormalities, it is a preliminary method that cannot provide a definitive histological diagnosis. The gold standard for diagnosing endometrial carcinoma is endometrial biopsy, which allows for the histological analysis of the sampled tissue. Endometrial biopsy is performed via hysteroscopy, an invasive technique that allows direct visualization of the endometrial cavity [23] and the removal of lesions such as polyps or small fibroids [19]. Hysteroscopy is an invasive examination that can be performed both in-patient and in an outpatient setting, with either general anesthesia or local anesthesia based on the patient’s characteristics. In most cases, patients tolerate the procedure [24] except for a small percentage who report it as a traumatic experience [19]. Additionally, though rare, complications such as bleeding, infection, and uterine injury may occur [25]. The failure rate of hysteroscopy, where the instrument cannot be successfully inserted into the uterine cavity, has been estimated at 4.2% [19]. It is important to highlight that obtaining an endometrial biopsy is not always straightforward. In fact, endometrial sampling fails in approximately 11% of cases (range 1–53%), primarily due to cervical stenosis [23]. Additionally, endometrial biopsy can be challenging, particularly in women with cervical stenosis, postmenopausal women, and those with hypertension or advanced age. In a multicenter cohort study of 356 women, the biopsy failure rate was significantly higher in nulliparous women compared to multiparous women (41.3% vs. 17.7%) [26]. However, emerging techniques such as vibrational biospectroscopy, including mid-infrared and Raman spectroscopy, are showing promising potential. These non-invasive methods generate a “biochemical fingerprint” to identify cancerous tissues, but the main limitation remains the requirement for a biological sample. Despite these innovations, biopsies are still essential for a definitive diagnosis [19].

For advanced-stage disease or when lymph node or metastatic involvement is suspected, advanced imaging techniques such as MRI or CT are employed. These modalities provide crucial information for staging and guiding treatment decisions. Furthermore, recent research into molecular biomarkers, including genetic mutations and epigenetic alterations, has opened up new avenues for less invasive diagnostic tests. The detection of specific proteins, mutations, or DNA methylation patterns in minimally invasive samples shows promise for improving the early diagnosis and prognosis of EC.

## 4. Innovative Diagnostic Approaches for Endometrial Cancer

Currently, there is no standardized screening program for endometrial cancer, either for the general population or for individuals considered at high risk [22]. The goal of screening is to detect atypical hyperplasia or endometrial cancer at the earliest possible stage to improve cure rates, reduce treatment-related morbidity, and lower mortality [19]. Given the importance of early detection in improving patient outcomes, there is a growing emphasis on developing screening methods that are both accessible and non-invasive. Traditional diagnostic methods, while effective, present significant limitations in terms of invasiveness and accessibility. This has driven recent interest in DNA methylation-based tests, which represent a novel, non-invasive alternative [27]. These DNA methylation-based tests represent a groundbreaking innovation in oncological diagnostics, offering a non-invasive solution with the potential to revolutionize early detection of endometrial cancer. These tests allow for the use of self-collected samples, reducing the need for invasive biopsy procedures and making the screening process more accessible and comfortable for patients. These tests leverage samples from biofluids like peripheral blood, uterine lavage, cervicovaginal fluid, and urine, offering new possibilities for early detection of endometrial cancer [28]. Epigenetic changes, particularly DNA methylation, play a crucial role in cancer development. These alterations can be detected even in the early stages of cancer progression, long before clinical symptoms appear. Each tumor cell can carry unique epigenetic markers, such as aberrant DNA methylation, which affect critical pathways involved in cell cycle regulation, signaling, tumor invasion, and metastasis [29]. DNA methylation is one of the most stable and detectable early changes in cancer cells, making it a highly reliable biomarker. While global hypomethylation contributes to chromosomal instability, hypermethylation of tumor suppressor genes leads to their transcriptional silencing, ultimately promoting carcinogenesis [30]. Focusing on these early and stable biomarkers, this review aims to evaluate minimally invasive diagnostic methods that leverage DNA methylation markers as alternatives to conventional biopsies. This review distinguishes itself by synthesizing data from recent studies conducted over the past decade (2013–2023), focusing on the latest advancements in minimally invasive and epigenetic-based diagnostic methods for endometrial cancer. Unlike previous reviews that have emphasized more invasive approaches, this analysis highlights novel, non-invasive methodologies, such as DNA methylation markers detectable in cervicovaginal and urine samples, as promising alternatives to traditional biopsy techniques. By focusing on these innovative, patient-friendly methods, this review aims to establish a foundation for expanding diagnostic options that are both practical and scalable, with the potential to enhance early detection and streamline clinical workflows. Recent studies show that methylation patterns are rapidly becoming a cornerstone in cancer diagnostics. In colorectal cancer (CRC), for instance, stool-based DNA methylation testing has markedly improved early detection and treatment outcomes [31]. Likewise, in endometrial cancer (EC), methylation analysis in urine provides a completely non-invasive method for detecting malignancy, presenting a promising tool for early diagnosis [32]. Moreover, given the invasive nature of endometrial biopsies, efforts are underway to explore alternatives, such as cervical scrapings, where cancer cells shed into the lower genital tract can be analyzed. This technique could emerge as a more patient-friendly, less invasive approach to detect EC while maintaining high diagnostic accuracy [33]. In addition to the mentioned methods, vaginal tampons and self-collected cervicovaginal samples represent further minimally invasive techniques that offer convenience and reduce the burden on healthcare systems [34]. These patient-centered approaches not only enhance compliance with screening programs but also represent a significant step forward in reducing the invasiveness and discomfort associated with traditional diagnostic methods. These approaches not only increase patient compliance with screening programs but also improve patient comfort and satisfaction.

## 5. Obesity and DNA Methylation in Endometrial Cancer (Pathogenetic Mechanisms of Obesity in Endometrial Cancer)

Obesity is an independent risk factor for the development of endometrial cancer. It is estimated that 70% to 90% of endometrial cancer patients are either overweight or obese. Obese women (with a BMI greater than 30 kg/m^2^) have a threefold higher risk due to elevated levels of circulating estrogen [35,36]. The meta-analysis by Crosbie et al. (2010) [8] examined the relationship between body mass index (BMI), hormone replacement therapy, and endometrial cancer risk. The findings indicated that each 5 kg/m^2^ increase in BMI raises the risk of endometrial cancer by 1.6 times. Women with a BMI of 42 kg/m^2^ have a ninefold higher risk compared to women of normal weight [8]. The underlying mechanisms linking obesity to endometrial cancer are primarily hormonal. Obesity creates a hyperestrogenic state because adipocytes convert androgens into estrogens, increasing overall estrogen production [37]. These elevated estrogen levels promote transcriptional activity and stimulate growth factor signaling pathways, which, in turn, encourage endometrial proliferation and may lead to hyperplasia and cancer [38]. Additionally, obesity is frequently accompanied by hyperglycemia and insulin resistance, both of which can dysregulate IGF-1 signaling and activate the mTOR pathway, driving increased cell proliferation [39]. Importantly, the metabolic environment in obese individuals contributes to the development of endometrial cancer not only through hormonal and metabolic effects but also via epigenetic modifications like DNA methylation. Chronic inflammation, elevated blood glucose, and insulin resistance disrupt normal DNA methylation patterns within endometrial tissue, impairing gene regulation and promoting carcinogenesis. Adipokines, which are cytokines secreted by adipose tissue, further exacerbate this issue by influencing DNA methylation. For instance, adipokines have been linked to the hypermethylation of genes like GJA1, a gene critical for intercellular communication. The hypermethylation of GJA1 impairs cellular function, thereby facilitating tumor development [9]. One of the most significant examples of obesity-related DNA methylation is the hypermethylation of the HAND2 gene. HAND2 acts as a critical regulator of estrogen-mediated endometrial growth, but in obese patients, this gene becomes hypermethylated, leading to the uncontrolled growth of endometrial tissue and accelerating cancer progression [40].

## 6. Emerging Epigenetic Biomarkers for EC Early Detection

**Figure 1:**
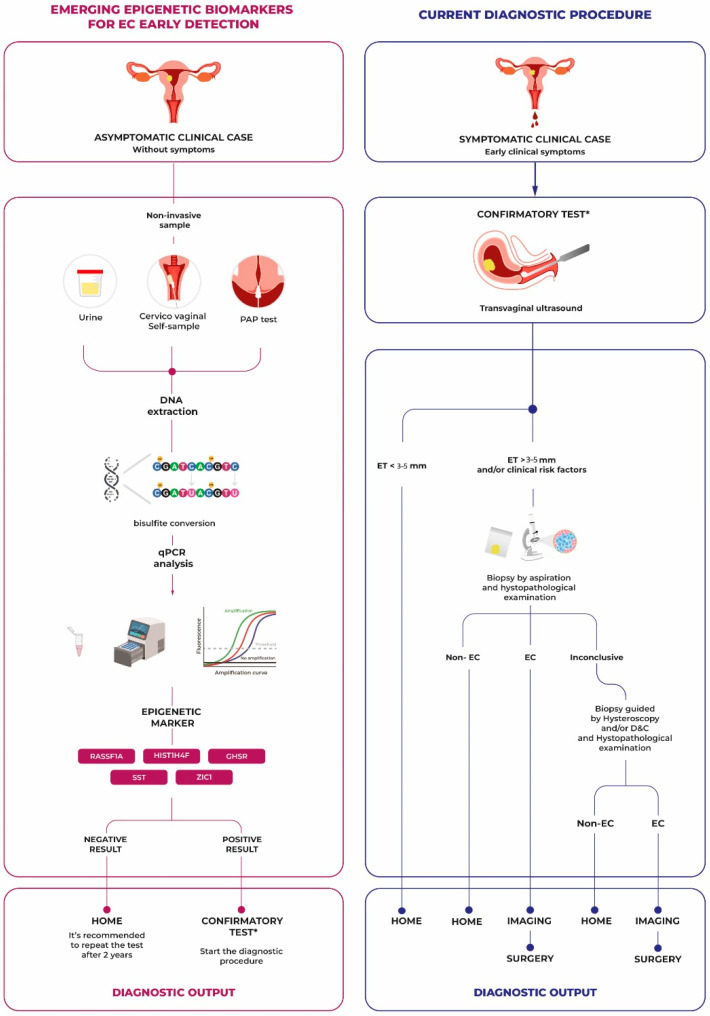
Emerging epigenetic biomarkers vs. current diagnostic procedures for early detection of endometrial cancer. This figure illustrates a comparison between conventional diagnostic pathways for symptomatic endometrial cancer (EC) and emerging biomarker-based methods for early detection in asymptomatic cases. On the left, non-invasive samples undergo DNA methylation analysis for epigenetic markers. A positive result prompts confirmatory diagnostic procedures, while a negative result suggests routine follow-up. On the right, the traditional diagnostic approach for symptomatic patients begins with transvaginal ultrasound, followed by biopsy if necessary. The integration of epigenetic biomarkers could lead to earlier, less invasive detection, potentially improving outcomes and reducing reliance on invasive procedures. * indicates that a positive result requires the initiation of diagnostic procedures. Different colors indicate different markers.

Recent advancements in endometrial cancer (EC) diagnostics have increasingly focused on minimally invasive specimen collection techniques and the identification of cancer-specific biomarkers. These innovations have shifted attention toward early detection methods, particularly through the analysis of DNA methylation signatures [41]. Aberrant DNA methylation in the promoter regions of tumor suppressor genes is recognized as a valuable biomarker for early-stage disease, given its role in gene silencing and the loss of tumor-suppressive functions. Recent studies demonstrate that endometrial cancer cells can be detected in vaginal and urine samples, as these cells are shed from the cervix, offering a convenient and cost-effective alternative to traditional pap test methods. Notably, tampon-based sampling has shown high specificity (96%) and sensitivity (82%) for detecting methylated DNA markers (MDM) in EC, supporting its potential as a reliable, patient-friendly collection method [42]. Additionally, research is exploring the use of peripheral blood samples for DNA methylation analysis, an approach known as ‘liquid biopsy’. This technique has the potential to identify circulating tumor DNA fragments in the blood, providing an even less invasive diagnostic option [43]. New self-administered endometrial sampling tools, such as micro-brushes, may further enable patients to collect samples at home, increasing adherence to screening programs [44]. In clinical studies, tampon-based sampling has shown a high sensitivity and specificity in detecting methylated markers such as CDH13, MLH1, and RASSF1A, further supporting its application as a non-invasive screening tool for EC [45]. Figure 1 illustrates the contrast between traditional diagnostic methods and emerging epigenetic approaches. Notably, DNA tests, which can detect circulating tumor-shed DNA without the need for intact tumor cells, are emerging as promising tools for triaging patients with postmenopausal bleeding and enabling faster, less invasive diagnosis [46]. The urgent need to improve EC diagnostics has spurred the development of novel approaches, combining minimally invasive cytological specimen collection with the detection of epigenetic alterations [47]. The analysis of methylated DNA in tampons for detecting endometrial cancer was first introduced in 2004. DNA methylation, a common epigenetic modification in cancer, involves adding a methyl group to CpG regions, commonly found in promoter regions of tumor suppressor genes. This process leads to the silencing of these genes, which lose their ability to suppress tumor growth. DNA methylation is particularly stable, making it detectable even in the early stages of carcinogenesis. Common techniques to detect methylation include bisulfite conversion followed by PCR or next-generation sequencing, allowing precise quantification of these epigenetic alterations [30,34,48]. Notably, DNA methylation in tumor suppressor genes differs from promoter hypermethylation linked to the inactivation of MLH1, MSH2, MSH6, or PMS2 genes in Lynch syndrome [49]. The selection of key methylation markers in this review, such as *CDH13*, *MLH1*, and *HS3ST2*, was based on their high diagnostic specificity, association with early-stage EC prognosis, and demonstrated accuracy in minimally invasive clinical applications, as shown in multiple recent studies [50]. In addition, several promising methylated DNA markers have been identified specifically in tampon-based collections for EC diagnosis, including CDH4, CYTH2, and DIDO1. These markers, along with EMX2OS, demonstrate diagnostic value due to their elevated specificity and sensitivity in detecting early-stage disease across multiple types of cancers, with significant implications for EC [42]. Furthermore, methylation patterns have shown subtype-specific profiles in EC. Recent analyses highlight how DNA methylation, combined with specific mutations such as PTEN loss and TP53 alterations, distinguishes endometrioids from serous subtypes, impacting prognosis and treatment responses uniquely [29]. Markers such as CDH13 and MLH1 are more frequently methylated in endometrioid endometrial carcinoma (EEC), providing diagnostic specificity for this subtype and highlighting potential differences in prognosis. MLH1 methylation, in particular, is associated with a significantly shorter disease-free survival (DFS) and overall survival (OS) in EEC patients, suggesting its value as a prognostic factor, especially for early-stage tumors [51]. To support this finding, a study highlighted the methylation status of MLH1 in EC patients, where immunohistochemistry (IHC) indicated MLH1 loss. A subsequent pyrosequencing analysis reported the methylation of the MLH1 promoter in DNA extracted from hysterectomy specimens [52]. The Cumulative Methylation Index (CMI), representing an aggregate measure of methylation across multiple tumor suppressor genes, has been found to be higher in EEC, further supporting its potential as a diagnostic tool [53,54]. Van den Helder et al. provide an overview of the reported DNA methylation markers for the detection of endometrial cancer (EC) in minimally invasive specimens. Among the 15 most promising markers identified are ADCYAP1, ASCL2, BHLHE22, CDH13, CDO1, CELF4, GALR1, HAND2, HS3ST2, HTR1B, MAGI2, MME, POU4F3, RASSF1, and ZNF662, with AUC values ranging from 0.80 to 0.96. The inclusion of emerging markers such as CDH4 and CYTH2 further supports the expanding range of methylation markers applicable in minimally invasive diagnostics for EC [33]. Along with CDO1, the test assessing methylation levels of CELF4 in cervical scraping samples can also serve effectively as a biomarker for EC in women with postmenopausal bleeding (PMB). The combined CDO1 and CELF4 test demonstrated high specificity as a supportive diagnostic tool, helping physicians distinguish between benign and malignant tumors in PMB patients and potentially reducing the need for invasive procedures [55]. These markers have shown potential for guiding early detection of EC in minimally invasive clinical settings [32,42,51,56]. Expanding on existing markers, recent studies have underscored the diagnostic and prognostic value of methylation in genes such as GSTP1, CDH1, and TIMP3, which are frequently silenced in advanced EC and can aid in risk stratification [57,58]. Similarly, RARβ and FHIT exhibit diagnostic potential due to their association with cancer invasiveness and cellular adhesion processes [59]. Emerging data on KLF4 and HS3ST2 also show their diagnostic relevance, with both genes exhibiting significant hypermethylation in endometrial cancer tissues compared to normal endometrium. KLF4, which plays a role in maintaining cell cycle stability, and HS3ST2, involved in cellular adhesion, have shown predictive accuracy with AUC values close to 0.95, suggesting their potential inclusion in biomarker panels for early detection [29,60]. In line with these findings, another analysis identified nine key methylation markers, including GHSR, SST, and ZIC1, with AUC values reaching 0.97 in urine and cervicovaginal samples [34]. These markers further expand the potential for non-invasive EC detection, making them valuable tools for clinical application. In addition, RASSF1A and HIST1H4F were identified as key DNA methylation markers in cervical pap brush samples, showing high diagnostic accuracy with AUC values of 0.938 and 0.951, respectively. These markers demonstrated strong potential for distinguishing EC from non-cancerous conditions, further enriching the pool of biomarkers for minimally invasive EC detection. Moreover, urine-based methylation testing for GHSR, SST, and ZIC1 demonstrated excellent discriminatory power, with AUC values of 0.95, 0.92, and 0.86, respectively, supporting its use as a non-invasive diagnostic approach [48]. A study identified hypermethylation in CD01, PITX2, and CDH13 in patients affected by early-stage EC, and the methylation level was compared to a control group and patients affected by endometrial hyperplasia. All genes resulted in having a high methylation level in EC. Moreover, EC patients with hypermethylation of CDH13 have not shown a complete response to conservative treatment, with respect to the rest of the EC cohort with a low methylation level of CDH13, suggesting his potential diagnostic and predictive value [61]. For a detailed summary of the most promising DNA methylation markers for the detection of EC, along with their diagnostic accuracy and sample types, refer to Table 1. Sample size inclusion and exclusion criteria are reported in the table caption. In conclusion, these DNA methylation markers represent promising tools for the early detection of endometrial cancer, with the potential to transform clinical practice through effective and non-invasive techniques. The integration of these markers into mass screening programs could not only improve patient prognosis by reducing the time to diagnosis but also reduce overall healthcare costs by minimizing the need for invasive procedures. Furthermore, the adoption of these tests in developing countries, where access to advanced healthcare facilities is limited, could have a significant impact on global public health.

## 7. Conclusions and Future Directions in Epigenetic Diagnosis of Endometrial Cancer

In conclusion, DNA methylation analysis has emerged as a promising tool for the early detection of endometrial cancer (EC), offering a non-invasive, cost-effective, and accurate alternative to traditional diagnostic methods. This review provides a unique perspective by highlighting minimally invasive sample collection techniques, such as urine and cervicovaginal samples and exploiting the potential of emerging epigenetic biomarkers. By focusing on accessible and patient-friendly diagnostic approaches, this work underlines an innovative path forward in EC diagnostics. Studies have demonstrated the high diagnostic accuracy of DNA methylation markers such as RASSF1A, HIST1H4F, GHSR, and SST in detecting EC across various sample types, including urine and cervicovaginal self-samples [30,32,34,48]. However, we focused our attention on whole methylated biomarkers identified in endometrial cancer (EC) as reported in the literature. Thus, methylated biomarkers detected in biopsies and resections were also included to expand the number of biomarkers that could be detected using a non-invasive diagnostic approach. Finally, a comprehensive methylation profile could improve early diagnosis and clinical outcomes for early-stage endometrial cancer. In particular, the use of non-invasive biological fluids is emerging as a promising strategy for the detection of hypermethylated biomarkers. These easily accessible biological fluids offer direct insight into the tumor circulating free DNA, which reflects the genetic alterations present in the tumor. Moreover, epigenetic signals are more stable in time, thus allowing for a more feasible detection in fresh samples. Recent studies have shown that the analysis of serum and urine samples can reveal specific epigenetic modifications, enabling not only the early diagnosis of endometrial cancer but also continuous disease monitoring [30,34,47]. The ability to use easily collected samples, such as those from the pap test or urine, represents a breakthrough in diagnostic approaches, reducing the need for invasive procedures and improving the sensitivity and specificity of screening. By providing less invasive options, these tests could improve patient compliance and facilitate early detection of endometrial cancer, especially in settings where traditional invasive procedures are difficult to implement. This review underscores the importance of these advancements by demonstrating how emerging biomarkers can transform patient screening practices and create a more effective, accessible pathway for early EC detection. Thus, a non-invasive approach, combined with the increasing precision of molecular analysis technologies, could significantly improve the management of endometrial cancer, facilitating early diagnosis and enhancing the prospects for effective treatment. Methylation tests using non-invasive procedures could also alleviate patients’ anxiety and discomfort, reducing the psychological burden often associated with traditional diagnostic methods. These markers not only distinguish malignant from benign conditions but also enable the early detection of EC, which is critical for improving patient outcomes. However, challenges remain, including the heterogeneity of study populations, variability in study designs, and the need for standardized methylation assays to ensure consistency across different clinical settings. Future research should focus on refining these biomarkers, enhancing their sensitivity and specificity, and exploring their integration into clinical practice. Large-scale clinical trials are essential to validate the utility of these markers in diverse populations and to establish standard protocols for their implementation in routine screening programs. Moreover, combining DNA methylation testing with other molecular biomarkers and imaging techniques could improve diagnostic accuracy and provide a more comprehensive understanding of EC progression. This multidisciplinary approach, incorporating both molecular and clinical expertise, is critical to ensuring that diagnostic strategies are robust, reproducible, and accessible to a wide range of patients. The adoption of epigenetic biomarkers as early and reliable indicators paves the way for a new era of preventive diagnosis for endometrial cancer, with practical large-scale application potential. Advances in epigenetic research will likely lead to the development of personalized diagnostic and therapeutic strategies, potentially revolutionizing the management of endometrial cancer. Continued exploration into the epigenetic mechanisms driving EC will open new avenues for early diagnosis, targeted treatment, and the prevention of disease recurrence. The future of EC diagnostics lies in a multidisciplinary approach that integrates cutting-edge molecular techniques with clinical expertise, ultimately transforming patient care.

## Figures and Tables

**Table 1:** Top DNA methylation markers for endometrial cancer detection. This table summarizes the most promising DNA methylation markers for EC detection, along with their AUC values and the type of sample used. These markers have shown a high capacity to distinguish EC from non-cancerous conditions, utilizing minimally invasive samples such as urine and cervical scrapes. On average, the samples collected were *n* = 95 and included women with histologically confirmed EC prior to receiving initial treatment. The inclusion criteria for patients encompassed age, histological grade, and type of EC.

DNA Methylation Marker	Sample Type	AUC Value	Diagnostic Role
RASSF1A	Cervical pap brush samples	0.94	High diagnostic accuracy for distinguishing EC from benign conditions [30].
HIST1H4F	Cervical pap brush samples	0.95	High accuracy in distinguishing EC from benign conditions [30].
GHSR	Urine	0.95	High discriminatory power in urine-based EC detection [34].
SST	Urine	0.92	Effective in detecting EC with self-collected urine samples [34].
ZIC1	Urine, cervicovaginal samples	0.86–0.97	Reliable in combination with other markers for accurate EC detection [32,34].
BHLHE22	Urine, cervicovaginal samples	Up to 0.97	High diagnostic accuracy in non-invasive samples [32,34].
CDH13	Urine, cervicovaginal samples	Up to 0.97	Enhances diagnostic sensitivity in non-invasive samples [48].
